# Contagion of Cholera

**Published:** 1867-08

**Authors:** R. L. Rea

**Affiliations:** Chicago


### CONTAGION OF CHOLERA.

By R. L. Rea, M.D., Chicago.

Theories are only absolutely perfect when they account for all the facts with which they are connected, and are valuable in proportion to their approach to this standard.

First, having a given number of facts, we form our theory, and all additional facts must agree with the theory thus formed.

Cholera has, seemingly, coquetted with the most assiduous researches of the wisest medical philosophers. Whether we consider its cause, nature, origin, or contagiousness, the resnlt is the same; they have been mocked by the echoes of their own eurekas. The heavens above, earth beneath, and the waters under it have been ransacked, and each in turn furnished a cause. Vegetable, animal, mineral, and aerial have borne their share of attributed influence. Each theory lasted long enough in the mind of its discoverer to assure him of its correctness, when a rough blast of facts demolished his beautiful fabric as easily as the north wind does the gossamer. If ever a disease was invented to take the conceit out of a man, that disease must have been cholera. Deny it if we will, the subject of cholera is involved in great obscurity in all its essential points. Its cause, nature, contagiousness, and treatment are matters of great uncertainty.

How opposite the class of persons affected. Two of the most temperate cases I was called to treat last year, I lost. Two of the most intemperate, irregular in habits and life, I saved. Every one's experience will show the same occasional result. As to localities affected by it, the village of Oxford, Ohio, 30 miles from Cincinnati, in one of the healthiest localities in the State, rolling, good water, and very good hygienic influence prevailing, in the last epidemic of cholera there died forty (40) out of a population of one thousand (1000), or 4 per cent.

Its treatment need simply be alluded to, from the most s entific to the most absurd, followed by success or failure wit. equal uncertainty, if you listen to the reports of those in whose hands cases fall. Do you believe that there were one hundred and two (102) cases of genuine cholera—consecutive cases—treated, without the loss of a single case? No more than I believe the reports of the homœopathic fraternity in Cincinnati, in the epidemic above alluded to, in which they reported, treated by themselves, 1400 cases—saved 1395!

The causes of these discrepancies are threefold:—1st. Want of knowledge. 2d. Faulty observation. 3d. Dishonesty. The first two are pardonable; the latter not. A man may not have all the knowledge he should, or draw wrong inferences from careless observation, or want of judgement; but he has no right to misrepresent.

My object, in the present brief article, is to present some suggestions, intended to harmonize some of the discrepancies regarding the contagiousness of cholera. We know how easy it is to prove by facts, either that cholera is or is not contagious. How a case brought into a neighborhood lights the fires of its ravages; and another brought in the same way is harmless as sunshine. How the devoted nurse is the victim of his own benevolence; and how another, going from house to house, in the midst of its most terrific forms, is a Daniel in the lion's den.

One fact, with reference to this disease, seems tolerably well settled, and that is, that whatever the exact nature of the predisposing cause, it spreads over the country, and whatever power we may have to prevent its localizing itself, by cleanliness and hygienic measures, the prime cause of cholera, or the epidemic influence if you please, is uncontrollable, that is, starts and spreads over the country, from one point to another, independent of hygienic influences, and exercises its power on the inhabitants of those regions over which it spreads; but when it does appear in any given locality, it is, in a measure at least, under the influence of prevalent circumstances, as air, water, cleanliness, and such as affect all diseases.

Poisons, as a class, are received into the blood, before exercising their toxic influence. Whether they will produce their poisonous influence, depends upon the power of the eliminating organs. If the poison is received in such quantities that they can throw it from the system as fast as received, no deleterious effects ensue; but when the poison is received so fast, or from any cause the excernent muscles are crippled so as to perform their functions imperfectly, the system suffers. Familiar examples of this are arsenic and opium. I believe this to be true, whether the poison enters through the stomach or lungs, and, furthermore, that in the fact before given, we have the explanation of many curious and difficult problems. Two persons exposed to the same influence with different results. My hospital colleague and myself visited the small-pex hospital in Cincinnati, were together the entire time during the visit, exposed to the same influences as far as possible; he came home and had varioloid; I escaped. We both received the poison into our systems—mine threw it off, his did not, with results as stated.

The familiar facts connected with dissecting wounds, is still further to the point. Dissecting wounds are very uncertain in their results; a very few cases result in poisoning the person inoculated. Some classes of subjects are especially virulent, but none are exempt from it, and there is little probability that of all the subjects received into the dissecting rooms, and the almost invariable injury to some connected with each class, that the infinitely small amount of poisoning can result from all those capable of infecting, in other words, that so little poisoning should come from so many exposures.

I have been, directly or indirectly, connected with dissecting rooms for eleven years; I was never harmed by the cadaver. Attending the wounded after the battle of Shiloh, I used my finger for a probe in the large holes made by the minie balls, and remember using my finger, which had the skin considerably broken, on an erysipelatous case, with not the slightest ill effect. Within two months after this, while operating on an arm, I pricked my finger, with a dissecting wound as the result—the patient dying with phlegmonous erysipelas, while I was suffering from the wound I had given myself. It seems hardly probable that this was the first time I was ever inoculated with poisonous virus, and still more so, when we consider the fact now alleged, that poison once on a broken surface is irretrievably in the system.

I do not think the condition of the system necessary to produce poisoning after its reception necessarily produces any feelings of conscious derangement in the individual, or that the eliminating organs are necessarily defective; but that the poison so received, as in my own case, may be overwhelming in quantity or virulence.

I think this theory of the influence of the poisons on the system *applied* will account for many things otherwise difficult to explain. Take the case of persons living constantly in the atmosphere of slaughter-houses, glue-factories, etc., and we wonder why they enjoy the perfect health they do. Their eliminating organs develop in power and function, if not in size, by use, and their constant labor to keep their decks clear, strengthens them in their arduous work.

In the case of children raised in squalor, filth, and effluvia, Chambers would say, dirt on their bodies keeps them warm, and so gives them additional vitality. But I think a better explanation would be, development of function by use.

Vaccination is supposed to exert some influence on the blood, by which the system is defended against visitations of small-pox. Would n't it be easier to account for it by attributing its influence by the power excited in the scavengers of the body, whereby they are enabled to do more effective duty, in ridding the system of variolous poison; and when overcome at all, are able to eliminate so much as to leave a very mild type of the original disease, the effect of the thoroughness of original medication. This theory, which I think will explain the facts concerning the contagiousness of cholera, is, that the epidemic influence spreads over a section of country—say Chicago. All breathe it. A given number, from the efficient power of the depurating organs, throw it off as fast as received into the system; others do not, and suffer from the disease. Those exposed may develop the disease by imprudence, when they would otherwise escape, adding the last hair to the camel's back by suddenly checking perspiration, or by indigestible food, or depressing excesses. This will hold true, not only of cholera, but to all diseases classed as contagious—measles, scarlatina, whooping-cough, etc. How irregular the contagion of such diseases is!

It is to this active, unceasing agency, resisting by its depurating power, that we are indebted for defence from disease *without*, as well as by generative poison *within*, the system.

